# Analysis of BDS Fractional Cycle Biases and PPP Ambiguity Resolution

**DOI:** 10.3390/s19214725

**Published:** 2019-10-31

**Authors:** Weiping Jiang, Wen Zhao, Hua Chen, Xuexi Liu, Xiangdong An, Qusen Chen

**Affiliations:** 1 School of Geodesy and Geomatics, Wuhan University, Wuhan 430079, China; 2009301610062@whu.edu.cn (W.Z.); hchen@sgg.whu.edu.cn (H.C.); xuexiliu@whu.edu.cn (X.L.); chenqs@whu.edu.cn (Q.C.); 2 GNSS Research Center, Wuhan University, Wuhan 430079, China; xdan@whu.edu.cn

**Keywords:** precise point positioning, Beidou navigation satellite system, ambiguity resolution, satellite-induced code bias, fractional cycle bias

## Abstract

It is difficult to enable traditional precise point positioning (PPP) with ambiguity resolution (AR) due to fractional cycle biases (FCBs), which limit the accuracy and reliability of positioning results. The BeiDou Navigation Satellite System (BDS) has been providing continuous positioning, navigation, and timing (PNT) services in the global region since the end of 2018. The BDS constellation includes geostationary earth orbit (GEO), inclined geostationary orbit (IGSO), and medium earth orbit (MEO) satellites. However, its hybrid constellation structure and the satellite-side multipath effect have hindered the BDS PPP AR for two main reasons: (1) some receivers have half-cycle biases between GEO and non-GEO satellites, which result in the inconsistency of hardware delays for each satellite type; (2) the correction model for elevation-dependent satellite-side multipath effect is only applicable to IGSO and MEO, while in the case of GEO the effect cannot be effectively weakened or eliminated. To rectify these problems, a suitable strategy is proposed for estimating BDS FCBs, whereby the GEO FCBs and non-GEO FCBs are estimated independently. Results show that the FCBs estimated by the new strategy for GEO and non-GEO are more stable compared to the traditional strategy. The GEO wide-lane (WL) FCBs fluctuate less than 0.3 cycle in one month, except for C05, while the variation of non-GEO WL FCBs is about 0.1 cycle. In addition, compared to the traditional strategy, the fractions of GEO WL ambiguities after the removal of FCBs estimated by the new strategy can be improved noticeably from 53.5% to 78.5%, and from 71.8% to 92.3% for <0.15 cycle and <0.25 cycle respectively, which could be comparable with non-GEO. Simultaneously, the improvement of GEO narrow-lane (NL) ambiguities is from 28.9% to 40.2%, and from 40.4% to 53.3% for <0.10 cycle and <0.15 cycle respectively, are less noticeable. This is mainly due to the low precision IGS products for GEO. After PPP AR, the mean convergence time is shorted from 56.0 min to 43.6 min, and from 71.6 min to 62.7 min for static PPP and kinematic PPP, respectively.

## 1. Introduction

Precise point positioning (PPP) can obtain high precision and absolute positioning based on precise satellite orbit and clock offset products [1]. The BeiDou Navigation Satellite System (BDS) has been providing global services since 27 December 2018, and the BDS satellites for precise positioning include geostationary earth orbit (GEO), inclined geostationary orbit (IGSO), and medium earth orbit (MEO). Due to the unique constellation design of BDS, the number of available satellites in the Asia-Pacific region is higher than in other areas. As we know, ambiguity resolution (AR) technology can shorten the convergence time of PPP and improve positioning accuracy and stability. However, the BDS hybrid constellation and the multipath effect in the satellite side significantly hinder ambiguity resolution, especially for PPP, which limits its application scope substantially. Many studies have found that hardware delays exist at the satellite and receiver terminals, which are highly coupled with the ambiguity parameters. It is very difficult to separate them from each other [2,3,4], leading to the loss of the integer property of ambiguity parameters. In relative positioning, the hardware delays can be eliminated by double-difference (DD), so the DD ambiguity has the integer characteristics. For PPP, it is necessary to restore integer characteristics for ambiguities by using satellite fractional cycle biases (FCBs) products [3] or precise clock offset products with FCBs corrections [4]. In addition, the ambiguity of PPP can also be fixed by using the decoupled clock model [5] or the zero-differenced hardware delays model [6]. In theory, these above PPP AR methods are also applicable to BDS.

However, some studies have found that there are some unique errors in the BDS satellite terminals, such as the severe multipath effect [7,8], which makes it difficult to fix the ambiguities for BDS PPP directly by using the above methods. Although the multipath of BDS MEO and IGSO satellites can be better weakened by the piecewise linear correction model based on the elevation angle [9], this model is not applicable to GEO. Because the elevation angle of a station-GEO pair is nearly unchanged, the deviation is an approximate constant which is difficult to be modeled. It is worth noting that the influence of the deviation on the observations is not the same for different station-GEO pairs. Therefore, the multipath effect at the GEO satellite end remains difficult to be effectively weakened or eliminated. Furthermore, the BDS GEO satellite signal is also affected by the multipath at the station, which shows a strong correlation between different days [10]. Moreover, for some commonly used receivers, such as the Trimble NETR9 and JAVAD TRE G3T DELTA, there is Inter-satellite-type bias (ISTB) between the BDS GEO satellites and the non-GEO satellites, which may cause half-cycle jumps for the observations of these receiver types [11,12]. For these reasons, we investigate the FCB estimation strategy of the BDS in depth, analyze the BDS ISTB and the satellite-side multipath effect in detail, and propose a suitable strategy for BDS FCBs estimation to achieve PPP fix solution.

After Section 1, the article is presented as follows: The BDS PPP model and FCBs estimation method are described in Section 2. Section 3 discusses the ISTB, BDS satellite multipath and BDS FCBs estimation strategies, and the characteristics of BDS FCBs in detail. Section 4 uses experiments to analyze the positioning accuracy and stability of the BDS static and kinematic PPP. Finally, conclusions are provided in Section 5.

## 2. Methods

The normal PPP model is described in the following equations [13,14]:

(1) $$P_{r,i}^{s}=\rho_{r}^{s}+c\left(dt_{r}-dt^{s}\right)+\frac{f_{1}^{2}}{f_{i}^{2}}I_{r,1}^{s}+T_{r}^{s}+d_{r,i}-d_{i}^{s}+\varepsilon_{P_{r,i}^{s}}$$

(2) $$L_{r,i}^{s}=\rho_{r}^{s}+c\left(dt_{r}-dt^{s}\right)-\frac{f_{1}^{2}}{f_{i}^{2}}I_{r,1}^{s}+T_{r}^{s}+\lambda_{i}\left(b_{r,i}-b_{i}^{s}\right)+\lambda_{i}N_{r,i}^{s}+\varepsilon_{L_{r,i}^{s}}$$

where r, s, i represent the receiver, satellite and frequency number, respectively; $f_{i}$, $\lambda_{i}$ donate the frequency i and wavelength; $\;dt_{r}$, $dt^{s}$ are the clock offsets of receiver and satellite in seconds; $I_{r,1}^{s}$ is the slant ionospheric delay at 1-frequency in meters; $T_{r}^{s}$ is the slant troposphere delay in meters; $d_{r,i}$,$\;d_{i}^{s}$ are the code hardware delays of receiver and satellite at the *i*-frequency in meters; $b_{r,i}$, $b_{i}^{s}$ are the phase delays of receiver and satellite at the *i*-frequency in cycles, respectively; $N_{r,i}^{s}$ is the integer ambiguity in cycles; $\varepsilon_{P_{r,i}^{s}}$, $\varepsilon_{L_{r,i}^{s}}$ are the measurement noises of pseudorange and carrier phase in meters, respectively. For simplicity, we assume that phase windup, phase center variation, relativistic effect, and tidal load have been corrected in Equations (1) and (2).

The ionospheric-free (IF) linear combination can be written as follows:

(3) $$P_{r,if}^{s}=\rho_{r}^{s}+c\left(dt_{r}-dt^{s}\right)+T_{r}^{s}+d_{r,if}-d_{if}^{s}+\varepsilon_{P_{r,if}^{s}}$$

(4) $$L_{r,if}^{s}=\rho_{r}^{s}+c\left(dt_{r}-dt^{s}\right)+T_{r}^{s}+\lambda_{if}\left(b_{r,if}-b_{if}^{s}\right)+\lambda_{if}N_{r,if}^{s}+\varepsilon_{L_{r,if}^{s}}$$

In PPP, the IGS precise clock products are generally used and the above equations could be re-written as follows:

(5) $$P_{r,if}^{s}=\rho_{r}^{s}+c\left(dt_{r}^{\prime }-dt^{\prime s}\right)+T_{r}^{s}+\varepsilon_{P_{r,if}^{s}}$$

(6) $$L_{r,if}^{s}=\rho_{r}^{s}+c\left(dt_{r}^{\prime }-dt^{\prime s}\right)+T_{r}^{s}+\lambda_{if}B_{r,if}^{s}+\varepsilon_{L_{r,if}^{s}}$$

(7) $$dt_{r}^{\prime }=dt_{r}+d_{r,if}/c,\;\;dt^{\prime s}=dt^{s}+d_{if}^{s}/c$$

(8) $$B_{r,if}^{s}=\frac{f_{1}}{f_{1}+f_{2}}B_{r,1}^{s}+\frac{f_{1}f_{2}}{f_{1}^{2}-f_{2}^{2}}N_{r,wl}^{s}$$

(9) $$B_{r,1}^{s}=N_{r,1}^{s}+b_{r,1}^{\prime s}$$

(10) $$b_{r,1}^{\prime s}=\frac{f_{1}}{f_{1}-f_{2}}\left(b_{r,1}-b_{1}^{s}\right)-\frac{f_{2}}{f_{1}-f_{2}}\left(b_{r,2}-b_{2}^{s}\right)-\frac{f_{1}+f_{2}}{f_{1}}\cdot \frac{d_{r,if}-d_{if}^{s}}{\lambda_{if}}$$

In Equations (7)–(10), the actual estimated IF ambiguity will absorb the pseudorange and phase hardware delay parameters, which is the reason for traditional PPP to only obtain float solutions.

In order to obtain the PPP fixed solution, it is necessary to separate the corresponding hardware delay parameters from the ambiguity parameters. The un-differenced ambiguity can be expressed as the following equation:

(11) $$B_{r,i}^{s}=N_{r,i}^{s}+b_{r,i}^{\prime s}$$

$B_{r,i}^{s}$ and $N_{r,i}^{s}$ represent the float and integer ambiguity in cycles, respectively;$\;b_{r,i}^{\prime s}=b_{r,i}^{\prime }-b_{i}^{\prime s}$; $b_{r,i}^{\prime }$, $b_{i}^{\prime s}$ are the actual estimated FCBs of receiver and satellite in cycles, respectively.

Generally, wide-lane (WL) ambiguity is fixed by the Melbourne-Wübbena (M-W) combination [15,16] using the following equations:

(12) $$B_{r,wl}^{s}=N_{r,wl}^{s}+b_{r,wl}^{\prime s}$$

(13) $$b_{r,wl}^{\prime s}=b_{r,wl}^{\prime }+b_{wl}^{\prime s}$$

(14) $$b_{r,wl}^{\prime }=(b_{r,1}-b_{r,2})-\frac{f_{1}-f_{2}}{f_{1}+f_{2}}\left(\frac{d_{r,1}}{\lambda_{1}}+\frac{d_{r,2}}{\lambda_{2}}\right)$$

(15) $$b_{wl}^{\prime s}=\left(b_{1}^{s}-b_{2}^{s}\right)-\frac{f_{1}-f_{2}}{f_{1}+f_{2}}\left(\frac{d_{1}^{s}}{\lambda_{1}}+\frac{d_{2}^{s}}{\lambda_{2}}\right)$$

It should be noted that the integer part of $b_{r,wl}^{\prime }$ cannot be separated from the ambiguity parameter, which is contained by the $N_{r,wl}^{s}$. But the effect of it will be balanced by narrow-lane (NL) FCB [3].

Assuming that *n* stations observe *m* visible satellites, for station *k* with *m* satellites, Equation (11) can be expressed as matrix in the following equation:

(16) $$\left[\begin{array}{c} B_{k,i}^{1}\\ B_{k,i}^{2}\\ \vdots \\ B_{k,i}^{m} \end{array}\right]=\left[I_{m\times m}\;R_{m\times 1}\;S_{m\times m}\right]\left[\begin{array}{c} N_{m\times 1}\\ b_{k,i}^{\prime }\\ b_{i\;m\times 1}^{\prime s} \end{array}\right],\;Q_{k,i\left(m\times m\right)}$$

where $I_{m\times m}$ donates the unit matrix; $R_{m\times 1}$ donates the *m* dimension column vector and all elements are 1; $S_{m\times m}$ is the *m* dimension diagonal matrix, all elements are −1; $N_{m\times 1}$ is the *m* dimension integer ambiguity column vector; $b_{k,i}^{\prime }$ is the FCB of receiver *k*; $b_{i\;m\times 1}^{\prime s}$ is the *m* dimension vector of satellites FCBs; $Q_{k,i\left(m\times m\right)}$ is the variance-covariance matrix.

The mathematical model of the FCB estimation has been discussed in detail in Section 2. However, many studies have found that there are some unique errors in BDS, such as the severe multipath effect in the satellite side and Inter-satellite-type bias, which may affect the accuracy of the BDS FCB. In Section 3, the characteristic of these biases will be analyzed.

## 3. BDS FCB Estimation

### 3.1. Inter-Satellite-Type Bias

Researches present that there are half-cycle jumps between GEO and non-GEO satellites for some receiver types. Thus, in this case, the DD ambiguities of GEO and non-GEO satellites for BDS zero-baseline cannot be fixed [11,12]. Due to different decoding methods of GEO and non-GEO signals in different receivers, ISTB may be introduced into the phase observations for BDS, which is related to frequency, receiver type and firmware version at the receiver side. For two receivers of the same type, ISTB can be eliminated by double-difference. But for two different types of receivers, ISTB may cause a half-cycle jump in DD ambiguity, which seriously damages its integer characteristic. Considering the influence of ISTB, observation Equations (1) and (2) can be rewritten as:

(17) $$P_{r,i}^{s}=\rho_{r}^{s}+c\left(dt_{r}-dt^{s}\right)+\frac{f_{1}^{2}}{f_{i}^{2}}I_{r,1}^{s}+T_{r}^{s}+d_{r,i}-d_{i}^{s}+\varepsilon_{P_{r,i}^{s}}$$

(18) $$L_{r,i}^{s}=\rho_{r}^{s}+c\left(dt_{r}-dt^{s}\right)-\frac{f_{1}^{2}}{f_{i}^{2}}I_{r,1}^{s}+T_{r}^{s}+\lambda_{i}\left(b_{r,i}^{\beta }-b_{i}^{s}\right)+\lambda_{i}N_{r,i}^{s}+\varepsilon_{L_{r,i}^{s}}$$

where $b_{r,i}^{\beta }=b_{r,i}+ISTB_{r,i}^{\beta }$; $ISTB_{r,i}^{\beta }$ is the ISTB at frequency i of receiver r in cycles; β is the number of satellite types for BDS. We assumed that the GEO satellites are type 1, and the non-GEO satellites are type 2. ISTB will be absorbed by $b_{r,i}^{\beta }$.

As we know, DD ambiguity can eliminate the influence of hardware delays perfectly. In order to show the influence of ISTB, a new analysis method is proposed for ISTB in this paper and DD-ambiguity is chosen as the research object. From the (8), (9), (10) and (12), the DD ambiguities are expressed in the following formulas:

(19) $$\lambda_{if}N_{r_{1}r_{2},if}^{\prime s_{1}s_{2}}=\frac{cf_{1}}{f_{1}^{2}-f_{2}^{2}}\left(N_{r_{1}r_{2},1}^{s_{1}s_{2}}+ISTB_{r_{1}r_{2},1}^{\beta_{1}\beta_{2}}\right)-\frac{cf_{2}}{f_{1}^{2}-f_{2}^{2}}\left(N_{r_{1}r_{2},2}^{s_{1}s_{2}}+ISTB_{r_{1}r_{2},2}^{\beta_{1}\beta_{2}}\right)$$

(20) $$N_{r_{1}r_{2},wl}^{\prime s_{1}s_{2}}=N_{r_{1}r_{2},wl}^{s_{1}s_{2}}+ISTB_{r_{1}r_{2},wl}^{\beta_{1}\beta_{2}}$$

(21) $$N_{r_{1}r_{2},nl}^{\prime s_{1}s_{2}}=\frac{f_{1}+f_{2}}{f_{1}}N_{r_{1}r_{2},if}^{\prime s_{1}s_{2}}-\frac{f_{2}}{f_{1}-f_{2}}N_{r_{1}r_{2},wl}^{\prime s_{1}s_{2}}$$

(22) $$N_{r_{1}r_{2},if}^{\prime s_{1}s_{2}}=N_{r_{1}r_{2},if}^{s_{1}s_{2}}+ISTB_{r_{1}r_{2},if}^{\beta_{1}\beta_{2}}$$

(23) $$ISTB_{r_{1}r_{2},i}^{\beta_{1}\beta_{2}}=\left(ISTB_{r_{1,i}}^{\beta_{1}}-ISTB_{r_{2,i}}^{\beta_{1}}\right)-\left(ISTB_{r_{1,i}}^{\beta_{2}}-ISTB_{r_{2,i}}^{\beta_{2}}\right)$$

Clearly, ISTB does not affect the DD ambiguity for two stations with the identical receiver type or the same observed satellite type. When it is an odd multiple of half-cycle, the fraction of $\;ISTB_{r_{1}r_{2},i}^{\beta_{1}\beta_{2}}$ is not equal to zero and $N_{r_{1}r_{2},i}^{\prime s_{1}s_{2}}$ equals $N_{r_{1}r_{2},i}^{s_{1}s_{2}}$ plus $ISTB_{r_{1}r_{2},i}^{\beta_{1}\beta_{2}}$, which will seriously destroy the integer characteristics of DD ambiguity. From the (19)–(23), it can be deduced that the difference between the actual estimated DD ambiguity $N_{r_{1}r_{2},i}^{\prime s_{1}s_{2}}$ and its true value $N_{r_{1}r_{2},i}^{s_{1}s_{2}}$ is as follows:

(24) $$\Delta_{ISTB,if}=N_{r_{1}r_{2},if}^{\prime s_{1}s_{2}}-N_{r_{1}r_{2},if}^{s_{1}s_{2}}=ISTB_{r_{1}r_{2},if}^{\beta_{1}\beta_{2}}=\frac{f_{1}^{2}}{f_{1}^{2}-f_{2}^{2}}ISTB_{r_{1}r_{2},1}^{\beta_{1}\beta_{2}}-\frac{f_{1}f_{2}}{f_{1}^{2}-f_{2}^{2}}ISTB_{r_{1}r_{2},2}^{\beta_{1}\beta_{2}}$$

(25) $$\Delta_{ISTB,wl}=N_{r_{1}r_{2},wl}^{\prime s_{1}s_{2}}-N_{r_{1}r_{2},wl}^{s_{1}s_{2}}=ISTB_{r_{1}r_{2},wl}^{\beta_{1}\beta_{2}}=ISTB_{r_{1}r_{2},1}^{\beta_{1}\beta_{2}}-ISTB_{r_{1}r_{2},2}^{\beta_{1}\beta_{2}}\;$$

(26) $$\Delta_{ISTB,nl}=N_{r_{1}r_{2},nl}^{\prime s_{1}s_{2}}-N_{r_{1}r_{2},nl}^{s_{1}s_{2}}=\frac{f_{1}+f_{2}}{f_{1}}\left(N_{r_{1}r_{2},if}^{'s_{1}s_{2}}-N_{r_{1}r_{2},if}^{s_{1}s_{2}}\right)-\frac{f_{2}}{f_{1}-f_{2}}\left(N_{r_{1}r_{2},wl}^{'s_{1}s_{2}}-N_{r_{1}r_{2},wl}^{s_{1}s_{2}}\right)\;=\frac{f_{1}+f_{2}}{f_{1}}\Delta_{ISTB,if}-\frac{f_{2}}{f_{1}-f_{2}}int\left(\Delta_{ISTB,wl}\right)$$

Only fractions of ISTB could be acquired from ambiguities, so all the possible ISTB values for GEO and non-GEO combination are $ISTB_{r_{1}r_{2},1}^{\beta_{1}\beta_{2}}=\left(-0.5,0,0.5\right)$,$\;ISTB_{r_{1}r_{2},2}^{\beta_{1}\beta_{2}}=\left(-0.5,0,0.5\right)$.

#### Analyses

The numerical analyses in this contribution are based on BDS observations collected with two different receiver types, namely Trimble and Javad. Two zero baselines were selected from IGS ZIM2-ZIM3 on DOY 150, 2015 and CUCC-CUTC on DOY 151, 2018, in Curtin University (http://saegnss2.curtin.edu.au/ldc/). The receiver information is listed in Table 1 and Table 2.

**Figure 1:**
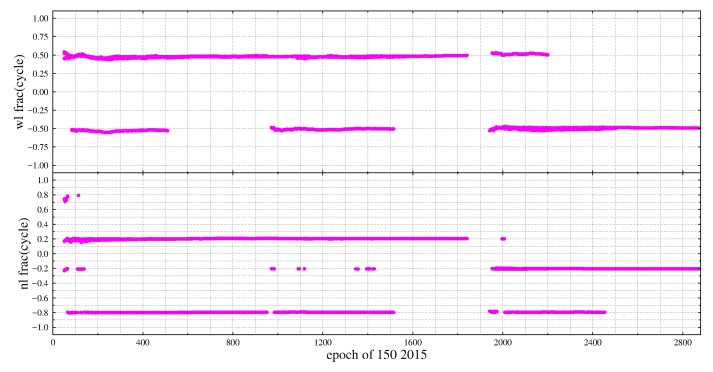
The fractional parts of WL and NL DD ambiguities for GEO and non-GEO satellites at zero-baseline ZIM2-ZIM3.

**Figure 2:**
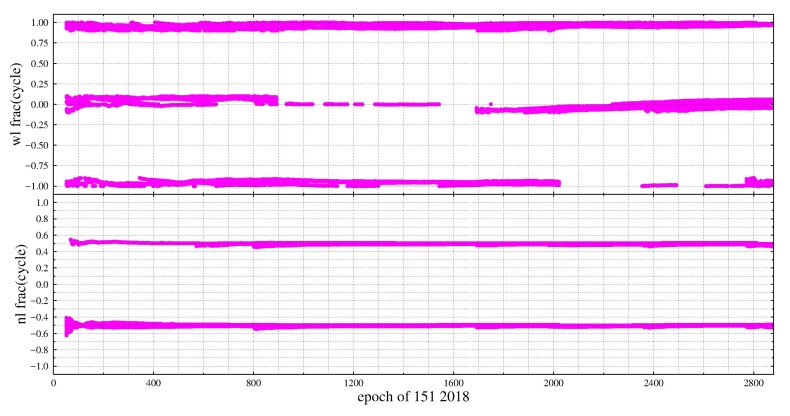
The fractional parts of WL and NL DD ambiguities for GEO and non-GEO Satellites at zero-baseline CUCC-CUTC.

According to Table 3, when $ISTB_{r_{1}r_{2},1}^{\beta_{1}\beta_{2}}$ equals ±0.5 and $ISTB_{r_{1}r_{2},2}^{\beta_{1}\beta_{2}}$ equals 0, $\Delta_{ISTB,wl}$ equals ±0.5. After $\Delta_{ISTB,wl}$ is rounded, we may get two possible values because of numerical error ε, so $\mathrm{int}\left(\Delta_{ISTB,wl}\right)=int\left(0.5\pm \varepsilon \right)=0\;or\;1$, $\Delta_{ISTB,nl}=\frac{f_{1}}{2\left(f_{1}-f_{2}\right)}\;or\;\frac{f_{1}-2f_{2}}{2\left(f_{1}-f_{2}\right)}$. Considering *f*_1_ = 1561.098, *f*_2_ = 1207.140,$\;\Delta_{ISTB,nl}\;=2.205\;or-1.205$ can be derived. In this case, the fractions of WL DD ambiguity between GEO and non-GEO satellites will be 0.5 cycle, while that of NL DD ambiguity will be −0.8, −0.2, 0.2 and 0.8 (Figure 1). Similarly, when $ISTB_{r_{1}r_{2},1}^{\beta_{1}\beta_{2}}=\pm 0.5\;\mathrm{and}\;ISTB_{r_{1}r_{2},2}^{\beta_{1}\beta_{2}}=\pm 0.5$, $\mathrm{int}\left(\Delta_{ISTB,wl}\right)=-1\;or\;1\;\mathrm{and}\;\Delta_{ISTB,nl}=\left(-0.5,0.5\right)$ could be derived from Table 4. That is, when $ISTB_{r_{1}r_{2},1}^{\beta_{1}\beta_{2}}$ and $ISTB_{r_{1}r_{2},2}^{\beta_{1}\beta_{2}}$ equal ±0.5, the fractional parts of NL DD ambiguity between GEO and non-GEO will be 0.5 cycle, while that of WL DD ambiguity will be an integer (Figure 2).

From Equation (11), if $ISTB_{r,i}^{1}$ is not equal to $ISTB_{r,i}^{2}$, GEO and non-GEO FCBs are different at receiver side, thus two FCB parameters should be considered. For Multi-GNSS Experiment (MGEX) networks, there are dozens of types and versions of receivers, among which some receivers may be $ISTB_{r,i}^{1}\neq ISTB_{r,i}^{2}$. Obviously, it is essential to accurately identify ISTB inconsistent receiver types from MGEX networks. However, this is not only onerous but is also unrealistic.

### 3.2. BDS Satellite-Induced Code Pseudorange Variations

Hauschild et al. found that the BDS pseudorange observations are unstable and have systematic deviation, which is strongly related to the satellite elevation angle [7,8]. Wanninger and Beer found that these code variations are elevation-dependent and there are no dependencies on receiver type, observation time, or satellite azimuth [9]. An elevation-dependent model was proposed for IGSO and MEO, which can weaken the fluctuation of IGSO/MEO pseudorange observations well. However, an absolute determination of the correction model is not feasible due to the ambiguous carrier phase observations and unknown signal delays, which will affect the FCBs [9]. This model is not applicable to the GEO, because the elevation angle of a GEO satellite relative to a certain station is basically unchanged, which is difficult to be modeled. Due to the relationship between the satellite-induced code variations of BDS and elevation, formulas are shown as:

(27) $$P_{r,i}^{s}=\rho_{r}^{s}+c\left(dt_{r}-dt^{s}\right)+\frac{f_{1}^{2}}{f_{i}^{2}}I_{r,1}^{s}+T_{r}^{s}+d_{r,i}-d_{i}^{s}+code\_bias_{P_{r,i}^{s}}\left(\mathrm{elev}\right)+\varepsilon_{P_{r,i}^{s}}$$

(28) $$L_{r,i}^{s}=\rho_{r}^{s}+c\left(dt_{r}-dt^{s}\right)-\frac{f_{1}^{2}}{f_{i}^{2}}I_{r,1}^{s}+T_{r}^{s}+\lambda_{i}\left(b_{r,i}^{\beta }-b_{i}^{s}\right)+\lambda_{i}N_{r,i}^{s}+\varepsilon_{L_{r,i}^{s}}$$

$code\_bias_{P_{r,i}^{s}}\left(\mathrm{elev}\right)$ is the satellite-induced code variation of receiver *r* with satellite *s* in meters.

(29) $$B_{r,wl}^{\prime s}=N_{r,wl}^{s}+b_{r,wl}^{\beta }-b_{wl}^{s}-code\_bias_{P_{r,nl}^{s}}\left(\mathrm{elev}\right)$$

(30) $$b_{r,wl}^{\beta }=\left(b_{r,1}^{\beta }-b_{r,2}^{\beta }\right)-\frac{f_{1}-f_{2}}{f_{1}+f_{2}}\left\{\frac{d_{r,1}-d_{1}^{s}}{\lambda_{1}}+\frac{d_{r,2}-d_{2}^{s}}{\lambda_{2}}\right\}$$

(31) $$code_{bias}{}_{P_{r,nl}^{s}}\left(\mathrm{elev}\right)=\frac{f_{1}-f_{2}}{f_{1}+f_{2}}\left\{\frac{code_{bias}{}_{P_{r,nl}^{s}}\left(\mathrm{elev}\right)}{\lambda_{1}}+\frac{code_{bias}{}_{P_{r,nl}^{s}}\left(\mathrm{elev}\right)}{\lambda_{2}}\right\}$$

**Figure 3:**
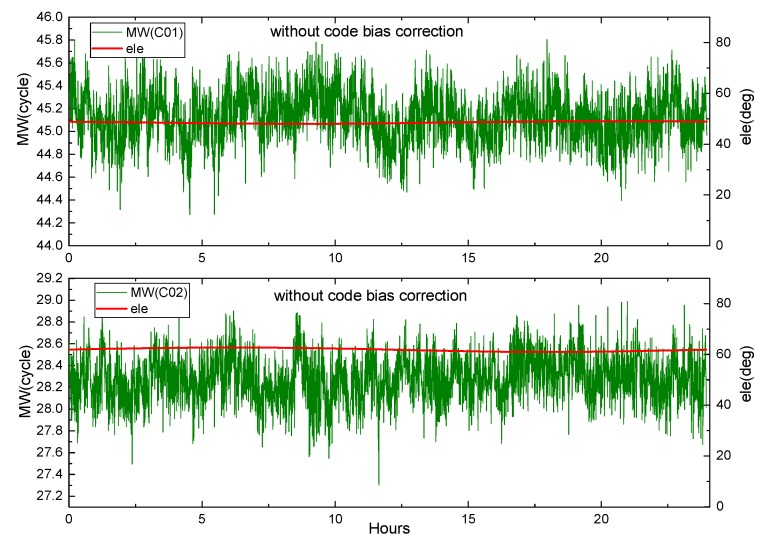
The M-W variation of C01, C02 at XMIS, on DOY 083, 2018.

**Figure 4:**
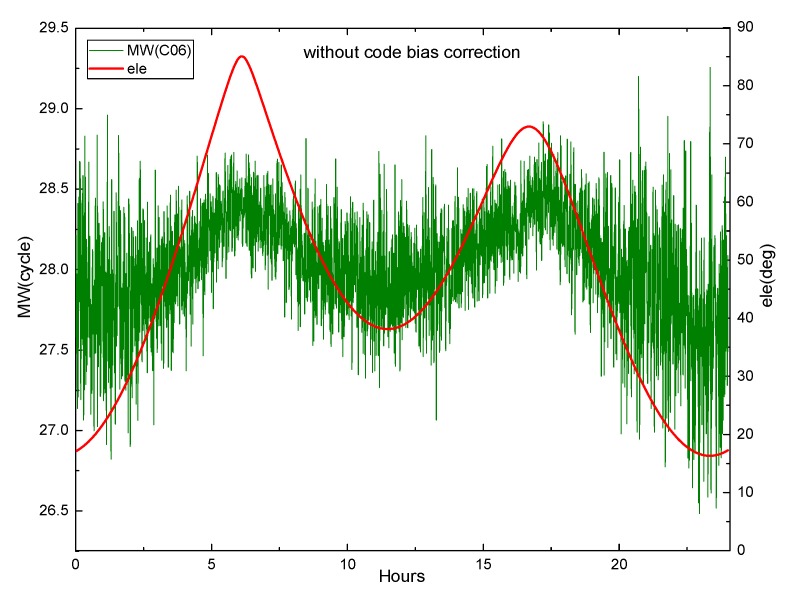
The M-W variation of C06 at XMIS, on DOY 083, 2018.

**Figure 5:**
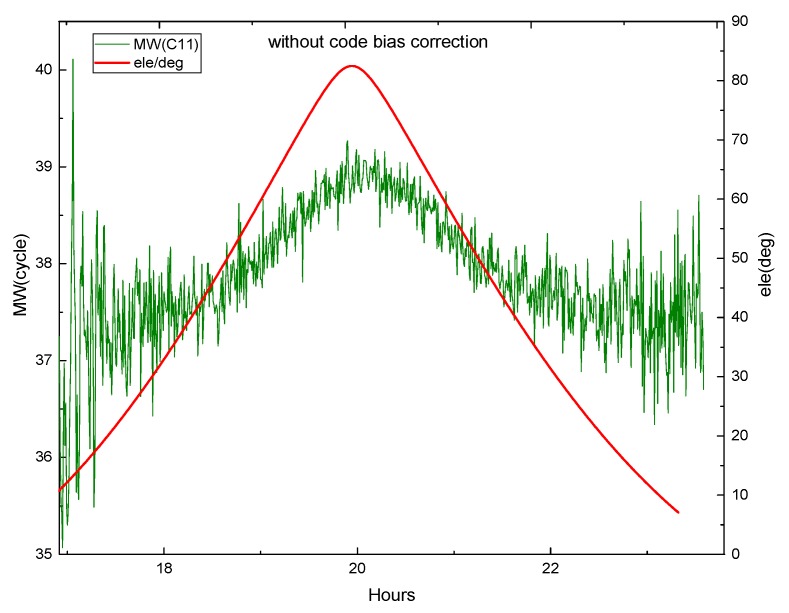
The M-W variation of C11 at XMIS, on DOY 083, 2018.

Equation (29) shows that the M-W observations will be affected by the pseudorange deviation. The effect is verified by M-W observations of B1 and B2 on DOY 083 2018 of MGEX station XMIS for the BDS. Figure 3 shows the variation of the M-W observations of C01 and C02 with elevation angle, while Figure 4 and Figure 5 show the variation of the M-W observations of C06 and C11.

From Figure 3, although the elevation of the BDS GEO satellite is almost unchanged, its M-W values show periodic fluctuations.

Figure 4 demonstrates that the fluctuation of the M-W value for C06 likes the shape of M, and the change of its elevation angle is also similar to M. They are very similar and highly correlated. In Figure 5, the M-W value of C11 has a very similar shape with its elevation angle. It can be seen that the M-W values of the BDS IGSO and MEO satellites are strongly correlated with elevation angles, which is mainly caused by the satellite-induced code pseudorange variations. Satellite-induced code variation is a unique bias in BDS, so it must be considered in BDS PPP AR.

**Figure 6:**
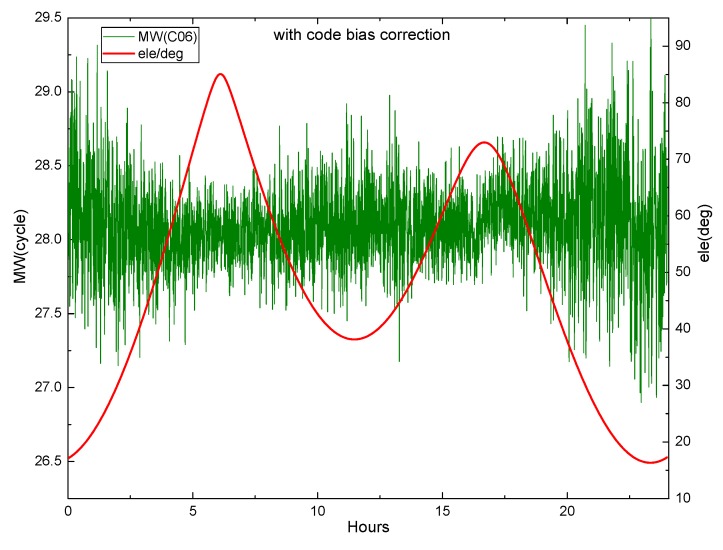
The corrected M-W variation of C06 at XMIS, on DOY 083, 2018.

**Figure 7:**
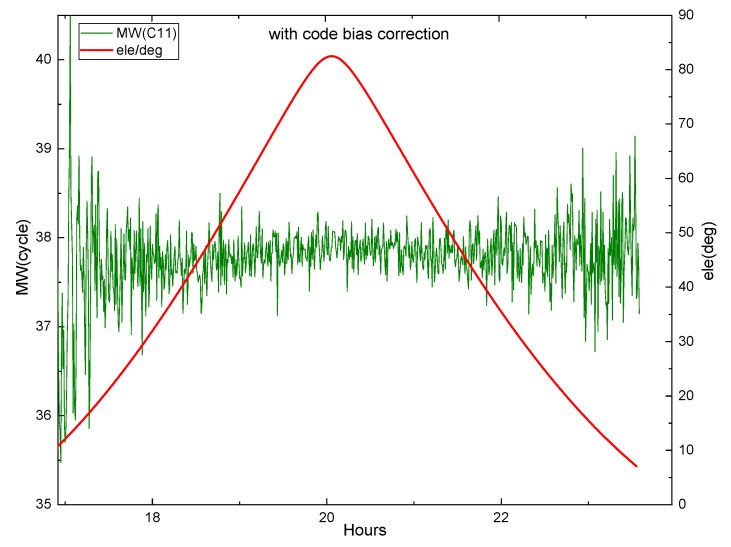
The corrected M-W variation of C11 at XMIS, on DOY 083, 2018.

The piecewise linear correction model proposed by Wanninger and Beer [9] is used to correct the multipath effect for BDS IGSO and MEO. However, this model is not applicable to GEO satellites. Figure 6 and Figure 7 display the corrected M-W values of C06 and C11 vary with the satellite elevation angle.

In Figure 6 and Figure 7, the corrected M-W values tend to be stable within an arc. However, it can be seen that at a low elevation angle, there are still some fluctuations, indicating that some errors still exist after the correction, and the code multipath effect has not been completely eliminated. Due to the limitation of the piecewise correction model, it is necessary to explore a more accurate model. Since there is no better correction model to be used, the piecewise model is used in this study.

### 3.3. Strategy Comparison

From the above analysis in Section 3.1 and Section 3.2, we can see that there are ISTB and pseudorange deviation problems in BDS. The ISTB results in the inconsistency of GEO and non-GEO hardware delay of some stations, which need to be estimated separately. For the BDS satellite-induced code variations, it has to be emphasized that an absolute determination of the correction models is not feasible due to the ambiguous phase observations and unknown signal delays. Hence, the corrections will affect the FCB estimations, which are essential for fixing ambiguity in PPP [9]. Meanwhile, because of the special orbit type of the GEO satellite, its elevation angle is almost unchanged, so the variation should be an approximate constant. But for different stations and different GEO, the elevation angles are not the same, so the variations are not the same consistency for different station-GEO pairs. Therefore, it will not be completely absorbed by the receiver or satellite hardware delay. In addition, the precision of BDS GEO products released by IGS is lower than that of IGSO and MEO.

**Figure 8:**
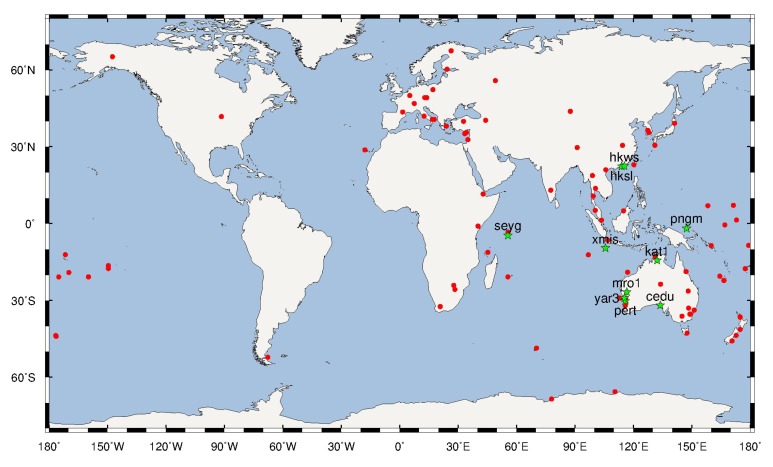
Distribution of MGEX network stations to calculate BDS FCB. The green stars represent the station selected to evaluate the accuracy.

Based on the above analyses, the paper proposes a new strategy for the classified estimation of BDS FCBs: GEO FCBs, and non-GEO FCBs. This strategy can effectively avoid ISTB problems, datum inconsistency introduced by the code variations correction model, and low precise GEO products, which can improve the accuracy of the BDS FCB. Meanwhile, we will call the unclassified estimation [14] the traditional strategy to avoid confusion. In order to verify the validity of the new strategy in this study, the BDS WL FCBs and NL FCBs are estimated by the new strategy and traditional strategy respectively. Then the results of the two strategies are compared and analyzed in detail. The data are collected by 103 stations from the MGEX network, on DOY 061-090, 2018. The distribution of the 103 MGEX stations is shown in Figure 8.

#### 3.3.1. BDS WL FCB

**Figure 9:**
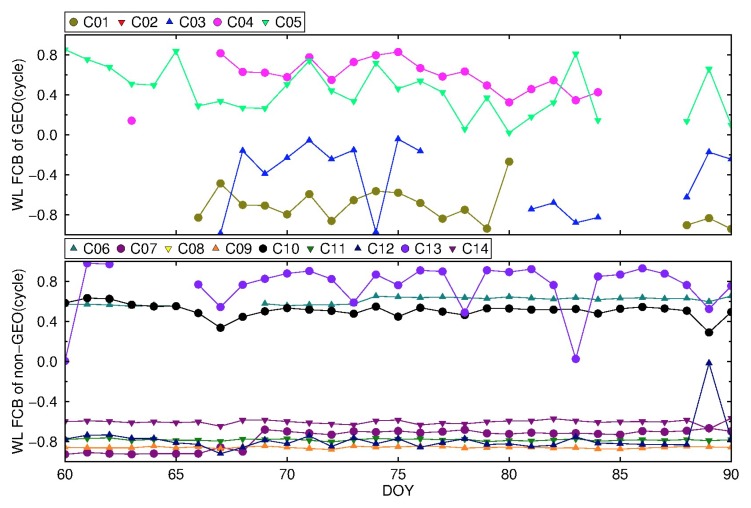
The variation of BDS WL FCB by the traditional strategy from DOY 061 to 090, 2018.

**Figure 10:**
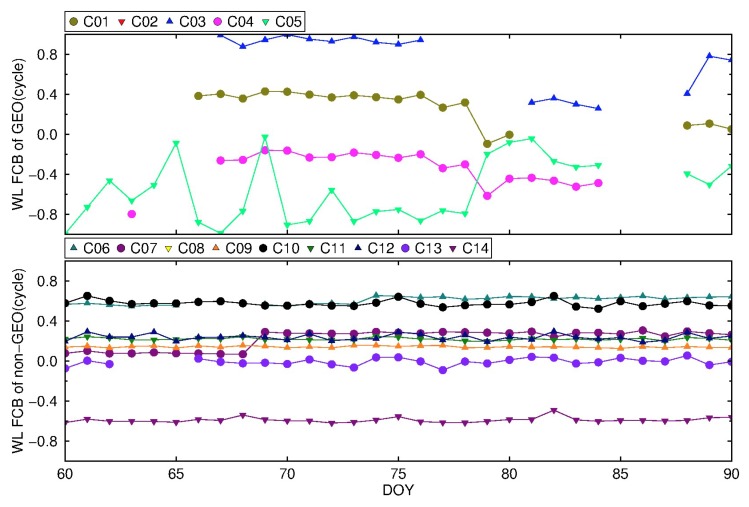
The variation of BDS WL FCB by the new strategy from DOY 061 to 090, 2018.

The BDS WL FCBs are calculated by a new strategy and traditional strategy based on the stations in Figure 8, with an interval of one day. The time series of WL FCBs are shown in Figure 9 and Figure 10. To eliminate FCBs datum, C02 and C08 are selected as reference satellites for GEO and non-GEO, respectively.

It can be seen from Figure 9 that within one month, the WL FCBs of IGSO and MEO estimated by the traditional strategy, except for C13, are relatively stable, and the fluctuation range is less than 0.2 cycle. The fluctuations are larger than 0.2 cycle for C13. On the other hand, GEO FCBs fluctuate greatly. From Figure 10, by the new strategy, the WL FCBs of IGSO and MEO are quite stable with fluctuations of less than 0.1 cycle. For GEO satellites, it is worth noting that the WL FCBs stability of C01, C03, C04 are comparable to that of IGSO and MEO in DOY 066-078, with fluctuations less than 0.1 cycle, but C05 still fluctuates greatly. For other time spans, GEO FCBs vary greatly. The reason for the instability may be the lack of IGS precision products for some GEO satellites during these days, resulting in insufficient GEO satellites that are actually used for FCBs estimation.

**Figure 11:**
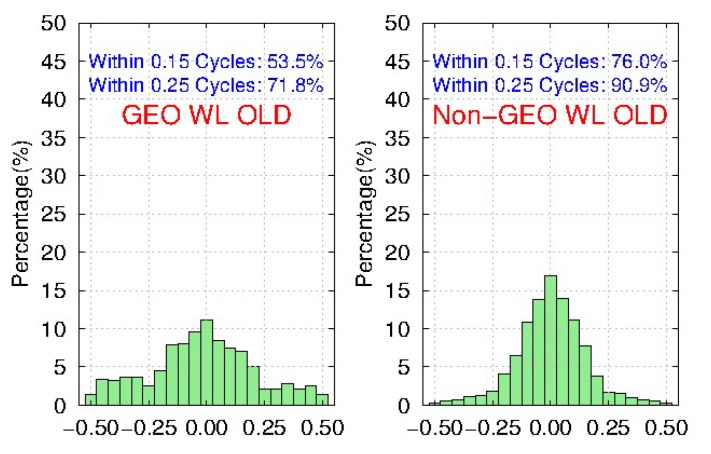
The distribution of BDS WL fractional parts after removal of FCBs estimated by the traditional strategy for 103 MGEX stations during DOY 061-090, 2018.

**Figure 12:**
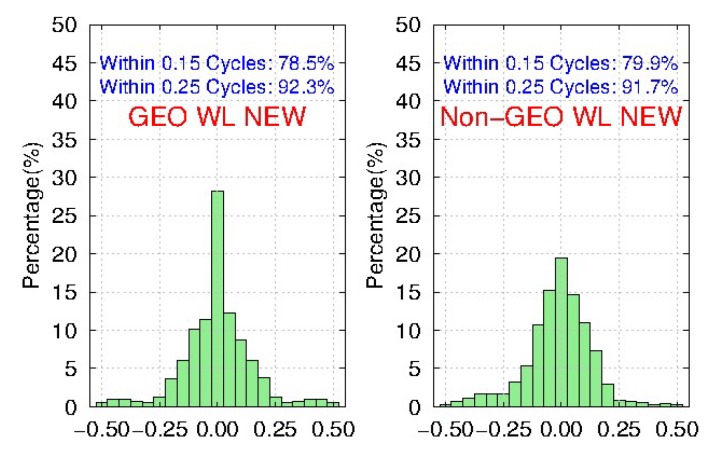
The distribution of BDS WL fractional parts after removal of FCBs estimated by the new strategy for 103 MGEX stations during DOY 061-090, 2018.

Meanwhile, Figure 11 and Figure 12 show the distribution of BDS WL fractional parts after the removal of FCBs by the traditional and new strategies, respectively. We find that compared to the traditional strategy, the ambiguity residuals of the new strategy are improved from 53.5% to 78.5%, and from 71.8% to 92.3% for <0.15 cycle and <0.25 cycle respectively for GEO, while the improvements for IGSO and MEO are very small. Furthermore, the precision of GEO WL FCBs could be comparable with IGSO and MEO in the new strategy. It shows that the new strategy can effectively improve the quality of BDS GEO FCBs.

#### 3.3.2. BDS NL FCB

**Figure 13:**
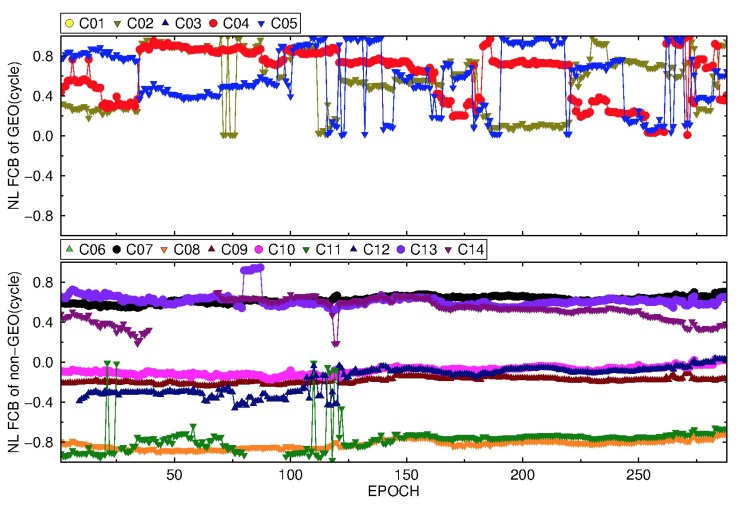
The variation of BDS NL FCB by the traditional strategy on DOY 083, 2018.

**Figure 14:**
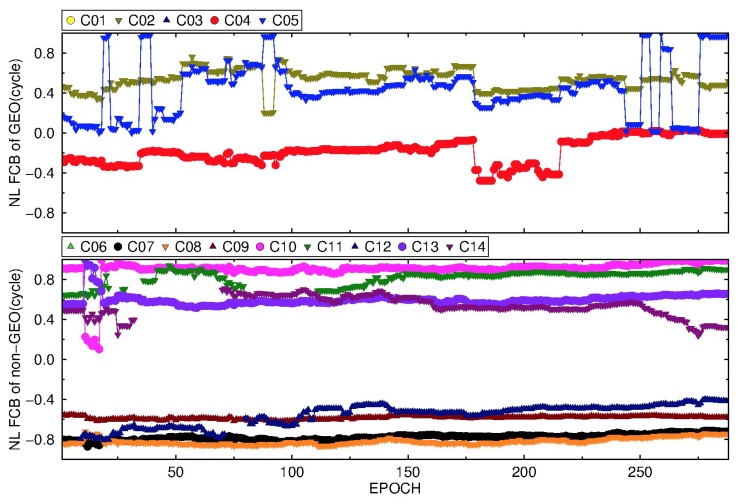
The variation of BDS NL FCB by the new strategy on DOY 083, 2018.

Because of the rapid variation of NL FCBs, it is estimated every 5 min during a day. It can be seen from Figure 13 and Figure 14 that the NL FCBs of IGSO and MEO are relatively stable except for C11, and the daily variation range of NL FCB is less than 0.2 cycles. The variation is very small for the adjacent 30–50 epochs (150–250 min), basically about 0.1 cycle or less. Compared to the IGSO and MEO, BDS GEO NL FCB is unstable, which is mainly due to the low precise IGS products for GEO. Moreover, the stability of BDS GEO NL FCB by the new strategy is still much better than that of the traditional strategy.

**Figure 15:**
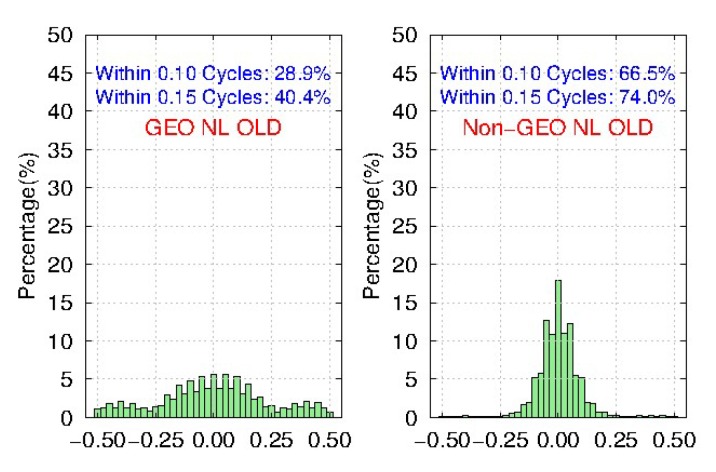
The distribution of BDS NL fractional parts after removal of FCBs estimated by the traditional strategy for 103 MGEX stations during DOY 061-090, 2018.

**Figure 16:**
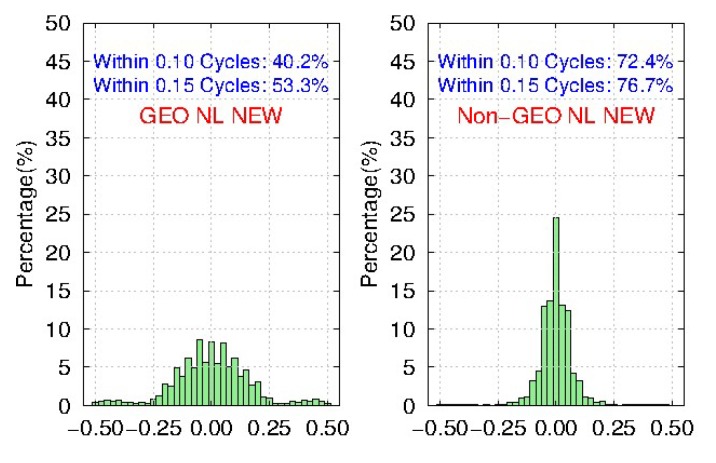
The distribution of BDS NL fractional parts after removal of FCBs estimated by the new strategy for 103 MGEX stations during DOY 061-090, 2018.

Figure 15 and Figure 16 show the distribution of BDS NL fractional parts after the removal of FCBs estimated by the traditional and the new strategy, respectively. Although it is not very obvious, there is still an improvement on the NL ambiguity residuals of IGSO and MEO by the new strategy. However, the NL ambiguity residuals of GEO by the new strategy are improved from 28.9% to 40.2%, and from 40.4% to 53.3% for <0.10 cycle and <0.15 cycle respectively, compared to the traditional strategy. The improvements of NL FCBs in the new strategy are not as obvious as that of WL FCBs. Besides, the precision of GEO NL FCBs is much worse than that of IGSO and MEO. It is mainly because of the low accuracy of current IGS products of GEO, which is worse than IGSO and MEO. What’s worse is that the wavelength of NL is only about 11cm, which is much shorter than WL, so GEO NL FCBs will be badly affected.

## 4. Experimental Results and Analysis

Using the new FCB strategy mentioned above, the BDS WL and NL FCBs were estimated to correct the fractional parts of BDS PPP ambiguities and obtain the fixed solutions. In this study, 10 stations distributed in the Asia-Pacific region (Figure 8) were selected to analyze and verify the accuracy of fixed solutions of the BDS PPP. The fastest time to first fix (TTFF) and positioning accuracy for PPP AR are analyzed. In this paper, convergence time is defined as the time required to achieve an accuracy of less than 5 cm, 5 cm, 10 cm for static PPP and less than 10 cm, 10 cm, 20 cm for Kinematic PPP in east, north and up directions, respectively. The TTFF is defined as the time taken for the first ambiguity to be successfully fixed [14]. The data duration is from DOY 061 to 090 in 2018, and the sample interval is 30 s. The estimated results are compared with the IGS weekly solutions.

### 4.1. Static PPP AR

**Figure 17:**
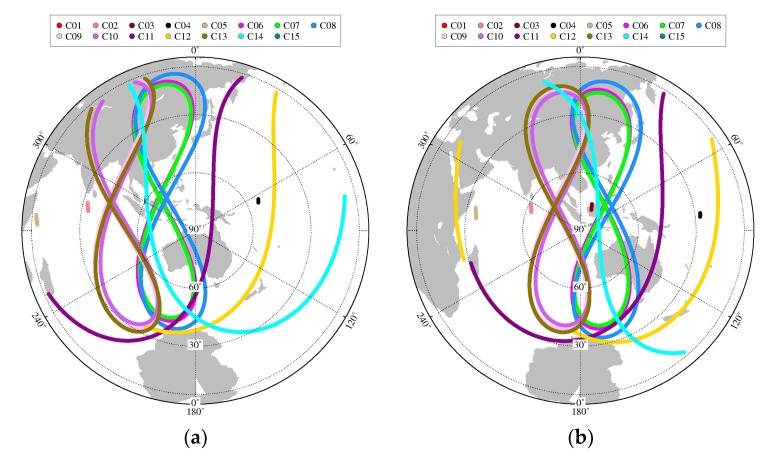
The skyplot of station: (**a**) KAT1; (**b**) XMIS.

**Figure 18:**
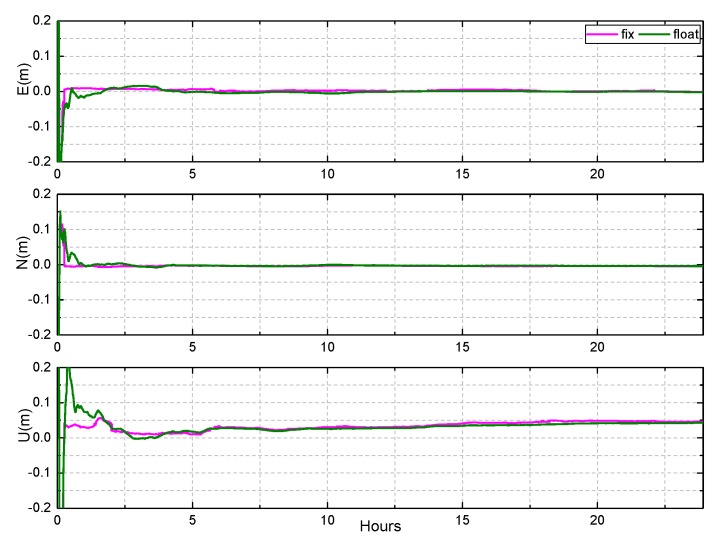
The results of BDS static PPP AR at KAT1, on DOY 083 of 2018.

**Figure 19:**
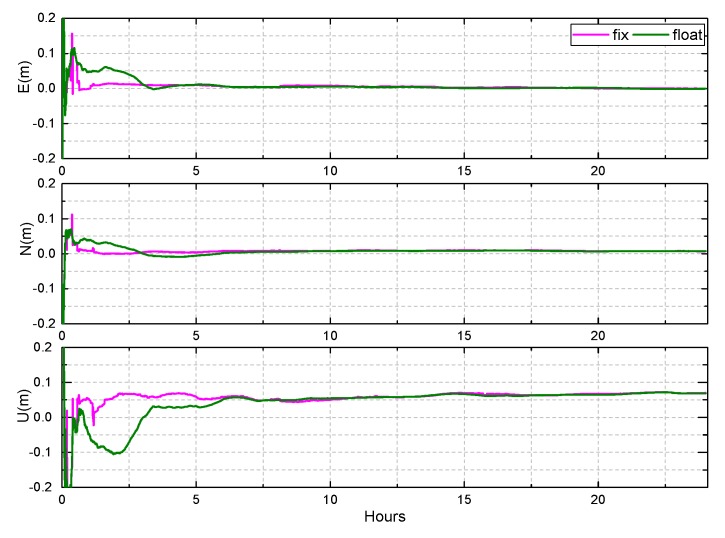
The results of BDS static PPP AR at XMIS, DOY 083 of 2018.

Due to large number of stations and long observation time, this paper chose the KAT1 and XMIS stations to display the results at DOY 083 of 2018 which are shown in Figure 17, Figure 18 and Figure 19.

The result of the fixed solution converges rapidly in east, north, and up directions when the ambiguity is fixed. After convergence, the horizontal accuracy of the fixed solution is less than 1 cm, while that of the float solution is approximately 2 cm. The fixed solution in the up direction is approximately 3 cm, while that of the float solution is approximately 4 cm. In addition, it can be seen that there is a small systematic deviation in the up direction, which may be due to no accurate phase center offset (PCO) and phase center variation (PCV) corrections of receiver antenna for BDS currently.

**Figure 20:**
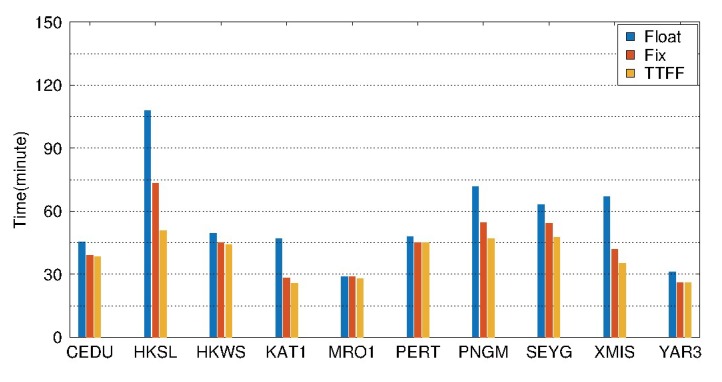
Average convergence time of float and fixed solutions, and TTFFs of 10 selected stations in static PPP processing from DOY 061 to 090, 2018.

Figure 20 and Table 5 show the results of BDS static PPP on DOY 061-091, 2018 for 10 selected stations in the Asia-Pacific region. As shown in Figure 20, the mean value of TTFFs for the 10 selected stations is about 40 min for static PPP AR. The convergence times are shortened for all the stations based on static PPP AR. The mean values of convergence time are 56.0 min, 43.6 min for float and fixed solution, respectively. The improvement is about 22.1%. Meanwhile, from Table 5 we can see that the RMS values of the fixed solution are less than 2 cm, 1 cm in the east, north directions. The accuracy of the north direction is the highest, followed by the east, while the up direction is the lowest for both float and fixed solutions. After the ambiguities are fixed, the accuracies are improved by about 16.7%, 23.0%, and 6.7% in the east, north, and up directions, respectively.

### 4.2. Kinematic PPP AR

**Figure 21:**
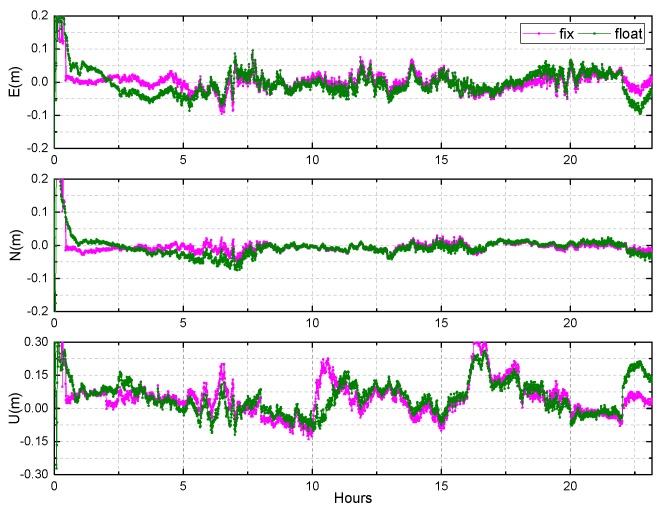
The results of BDS kinematic PPP AR at KAT1, on DOY 083 of 2018.

**Figure 22:**
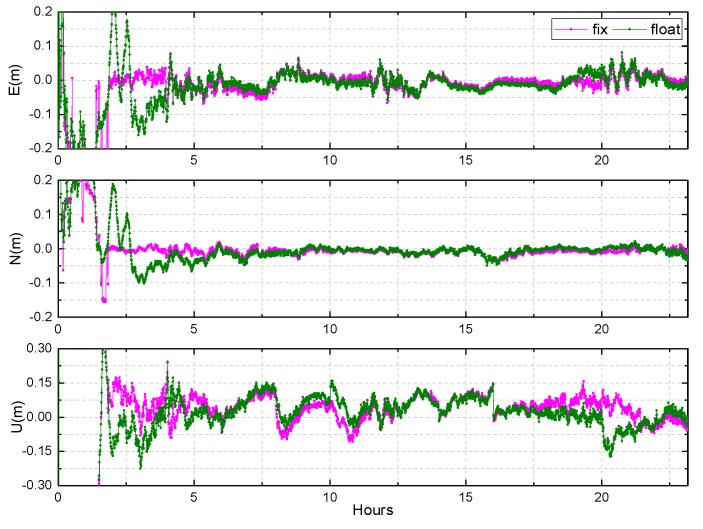
The results of BDS kinematic PPP AR at XMIS, DOY 083 of 2018.

The observations of 30 days from 10 stations in Asia-Pacific are used to evaluate the BDS kinematic PPP. The results of XMIS and KAT1 on DOY 083, 2018 are shown in Figure 21 and Figure 22.

The convergence time is clearly shortened when the ambiguities are fixed, especially in the east and north directions. The RMS of position error can be improved from 6 cm, 4 cm to 5 cm, 3.5 cm in the east and north directions, respectively. In the up direction, the accuracy of the fixed solution is slightly better than that of float solution after convergence, but the improvement is not obvious. Furthermore, the precision of the up direction is poor, and the jitter after convergence is significant, exceeding 20 cm at times. This may be due to no accurate PCO and PCV corrections of the receiver antenna for the BDS and the current configuration of the BDS constellation.

**Figure 23:**
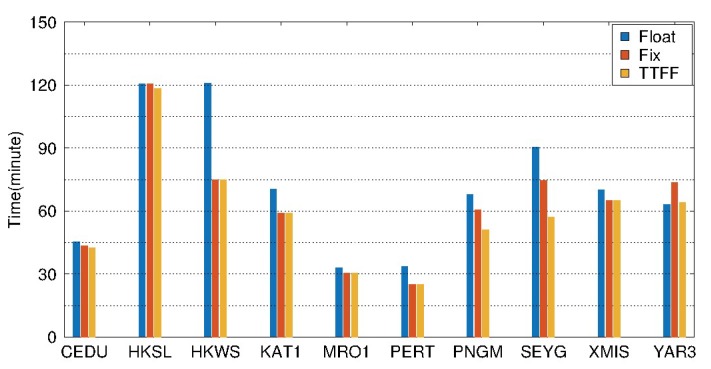
Average convergence time of float and fixed solutions, and TTFFs of 10 selected stations in kinematic PPP processing from DOY 061 to 090, 2018.

The results of BDS kinematic PPP during DOY 061-091, 2018 are shown in Figure 23 and Table 6 for 10 selected stations in the Asia-Pacific region. As shown in Figure 23, the average TTFF is about 58.7 min for kinematic PPP AR. Except for the station YAR3, the convergence time is shortened for all the other stations after kinematic PPP AR. The mean convergence times are 71.6 min and 62.7 min for float and fixed solution, respectively. The improvement is about 12.4%. From Table 6, it can be seen that the RMS values of the fixed solution are less than 5 cm, and 3.5 cm in the east, north directions. After the ambiguities are fixed, the accuracies are improved by about 18.1%, 21.2% and 7.9% in the east, north and up directions, respectively. The improvement of accuracy in the east and north directions is very obvious, while the improvement is slight in the up direction.

## 5. Conclusions

In this paper, several BDS biases are studied and analyzed in detail to enable ambiguity resolution. Research shows that there are half-cycle jumps between BDS GEO and non-GEO satellites for some receiver types, which results in inconsistencies in hardware delay between GEO and non-GEO for these receivers. Furthermore, there are serious satellite-induced code biases for the BDS satellites. The biases in IGSO and MEO can be corrected with existing models, while there is no correction model available to GEO.

In order to obtain high precision FCBs, this paper proposes a new strategy for estimating BDS GEO and non-GEO FCBs, respectively. This strategy could effectively avoid ISTB problems. Results show that the GEO and non-GEO FCBs estimated by the new strategy are more stable compared to the traditional strategy. The variation of GEO FCBs is less than 0.3 cycle in one month, except for C05. The FCBs of non-GEO satellites in one month have fluctuated 0.1 cycle. In addition, compared to the traditional strategy, the fractions of GEO WL ambiguities after removal of FCBs estimated by new strategy can be obviously improved from 53.5%, 71.8% to 78.5%, 92.3% for <0.15 cycle and <0.25 cycle respectively, which could be comparable with non-GEO. Simultaneously, the improvement of the GEO NL ambiguities is from 28.9%, 40.4% to 40.2%, 53.3% for <0.10 cycle and <0.15 cycle, which is not very obvious. This is because the current IGS products for GEO are in low precision. However, the NL FCBs of the adjacent 10–20 epochs (50–100 min) are less than 0.1 cycle. The daily variation of IGSO and MEO NL FCBs is much smaller, less than 0.2 cycle, except for C13. The variation of the adjacent 30–50 epochs (150–250 min) is very small, being less than 0.1 cycle.

In this study, the fixed solution of the BDS PPP is analyzed by using 10 stations distributed in the Asia-Pacific region. The results show that the convergence speed is faster when ambiguities are fixed. The accuracy and stability of static BDS PPP AR in the east, north, and up directions are improved compared with float solutions. The mean convergence times are shortened from 56.0 min to 43.6 min, and from 71.6 min to 62.7 min for static and kinematic PPP AR, respectively. The east and north directions are improved greatly, while the up direction is improved slightly. For static PPP, the position biases of ambiguity-fixed PPP are improved by about 16.7%, 23.0%, and 6.7% in the east, north, and up directions, respectively. Similarly, the improvements are approximately 18.1%, 21.2%, and 7.9% in the east, north, and up directions respectively, for BDS kinematic PPP.

## Figures and Tables

**Table 1:** Receiver information of ZIM2 and ZIM3 on DOY 150, 2015.

Station	Receiver Type	Version
ZIM2	TRIMBLE NETR9	4.85
ZIM3	TRIMBLE NETR9	4.93

**Table 2:** Receiver information of CUCC and CUTC on DOY 151, 2018.

Station	Receiver Type	Version
CUCC	JAVAD TRE_G3T DELTA	3.73
CUTC	TRIMBLE NETR9	5.30

**Table 3:** Frequency 1 with half-cycle jump (Unit: cycle).

$ISTB_{r_{1}r_{2},1}^{\beta_{1}\beta_{2}}$	$ISTB_{r_{1}r_{2},2}^{\beta_{1}\beta_{2}}$	$\Delta_{ISTB,wl}$	$int\left(\Delta_{ISTB,wl}\right)$	$\Delta_{ISTB,if}$	$\Delta_{ISTB,nl}$
0.5	0	0.5	0 or 1	$\frac{f_{1}^{2}}{2(f_{1}^{2}-f_{2}^{2})}$	$\frac{f_{1}}{2\left(f_{1}-f_{2}\right)}or\frac{f_{1}-2f_{2}}{2\left(f_{1}-f_{2}\right)}$
−0.5	0	−0.5	0 or −1	$\frac{-f_{1}^{2}}{2(f_{1}^{2}-f_{2}^{2})}$	$\frac{-f_{1}}{2\left(f_{1}-f_{2}\right)}or\frac{-f_{1}+2f_{2}}{2\left(f_{1}-f_{2}\right)}$

**Table 4:** Frequency 1 and 2 all with half-cycle jump (Unit: cycle).

$ISTB_{r_{1}r_{2},1}^{\beta_{1}\beta_{2}}$	$ISTB_{r_{1}r_{2},2}^{\beta_{1}\beta_{2}}$	$\Delta_{ISTB,wl}$	$int\left(\Delta_{ISTB,wl}\right)$	$\Delta_{ISTB,if}$	$\Delta_{ISTB,nl}$
0.5	0.5	0	0	$\frac{f_{1}}{2\left(f_{1}+f_{2}\right)}$	0.5
0.5	−0.5	1	1	$\frac{f_{1}}{2\left(f_{1}-f_{2}\right)}$	0.5
−0.5	0.5	−1	−1	$\frac{-f_{1}}{2\left(f_{1}-f_{2}\right)}$	−0.5
−0.5	−0.5	0	0	$\frac{-f_{1}}{2\left(f_{1}+f_{2}\right)}$	−0.5

**Table 5:** The average accuracy of BDS static PPP for 10 stations (Unit: cm).

Direction	Fix	Float	Accuracy Improvement (%)
E	1.79	2.15	16.7
N	0.97	1.26	23.0
U	5.81	6.23	6.7

**Table 6:** The average accuracy of BDS kinematic PPP for 10 stations (Unit: cm).

Direction	Fix	Float	Accuracy Improvement (%)
E	4.80	5.86	18.1
N	3.13	3.97	21.2
U	12.17	13.21	7.9
